# Anc1, a Protein Associated with Multiple Transcription Complexes, Is Involved in Postreplication Repair Pathway in *S. cerevisiae*

**DOI:** 10.1371/journal.pone.0003717

**Published:** 2008-11-13

**Authors:** Rachel L. Erlich, Rebecca C. Fry, Thomas J. Begley, Danielle L. Daee, Robert S. Lahue, Leona D. Samson

**Affiliations:** 1 Department of Biology, Massachusetts Institute of Technology, Cambridge, Massachusetts, United States of America; 2 Center for Environmental Health Sciences, Massachusetts Institute of Technology, Cambridge, Massachusetts, United States of America; 3 Department of Biological Engineering, Massachusetts Institute of Technology, Cambridge, Massachusetts, United States of America; 4 Eppley Institute for Research in Cancer and Allied Diseases, University of Nebraska Medical Center, Omaha, Nebraska, United States of America; University of Minnesota, United States of America

## Abstract

Yeast strains lacking Anc1, a member of the YEATS protein family, are sensitive to several DNA damaging agents. The YEATS family includes two human genes that are common fusion partners with *MLL* in human acute leukemias. Anc1 is a member of seven multi-protein complexes involved in transcription, and the damage sensitivity observed in *anc1*Δ cells is mirrored in strains deleted for some other non-essential members of several of these complexes. Here we show that *ANC1* is in the same epistasis group as *SRS2* and *RAD5*, members of the postreplication repair (PRR) pathway, but has additive or synergistic interactions with several other members of this pathway. Although PRR is traditionally divided into an “error-prone” and an “error-free” branch, *ANC1* is not epistatic with all members of either established branch, and instead defines a new error-free branch of the PRR pathway. Like several genes involved in PRR, an intact *ANC1* gene significantly suppresses spontaneous mutation rates, including the expansion of (CAG)_25_ repeats. Anc1's role in the PRR pathway, as well as its role in suppressing triplet repeats, point to a possible mechanism for a protein of potential medical interest.

## Introduction

Understanding the role of all genes that function to provide resistance upon chemical exposure will provide a systems level view of how cells respond to changing environments, and an understanding of what happens to the cell and the organism when this system is impaired. Recently, we screened all of the non-essential yeast genes to identify those that provide resistance to DNA damaging agents [1], [2]. Based on the genes of known function whose deletion resulted in sensitivity, we identified several unexpected cellular processes that were overrepresented among damage sensitive mutants [1], [2]. RNA polymerase II (Pol II)-mediated transcription was among the many pathways that were significantly overrepresented [1], [2].

The product of the *ANC1* gene (also known as *TAF14* and *TFG3*) is a member of at least seven multi-protein complexes that have distinct but related cellular roles, the common theme being their involvement in RNA Polymerase II-mediated transcription. Anc1-containing complexes include two members of the RNA Pol II holoenzyme, TFIID and TFIIF, the chromatin remodeling complexes RSC, SWI/SNF, and INO80, the histone acetyltransferase complex, NuA3 and the transcriptional activation adapter complex Mediator. [3]–[9]. The sensitivity of *anc1*Δ *S. cerevisiae* strains to UV light, γ-irradiation the DNA alkylating agents methane methylsulfonate (MMS) and 4-nitroquinoline 1-oxide (4NQO), and to hydroxyurea (HU) was recently reported [1], [2], [8], [10]. How Anc1 promotes survival after exposure to DNA damage and replicative stress has not, until now, been explored.

The Anc1 protein contains a highly conserved YEATS domain that is present in three yeast (Yaf9, Anc1 and Sas5) and four human (ENL/MLLT1, AF9/MLLT3, GAS41 and YEATS2) proteins. Three of the four YEATS family human proteins are associated with the human mixed linkage leukemia gene: *MLL* gene fusions occur in ∼3% of AML (acute myeloid leukemia) and 8–10% of ALL (acute lymphoid leukemia) [11]. Both *ENL* and *AF9* are common translocation partners with *MLL* in these cancers, and GAS41 has been shown to interact directly with the product of the *AF10* gene, another *MLL* fusion partner [12]. Together, *ENL*, *AF9* and *AF10* fusions with *MLL* account for about 35% of spontaneous human acute leukemias with *MLL* gene fusions [11]. The function of the YEATS domain is still largely unknown, although it was recently reported that tagged ENL binds specifically to histones H1 and H3 via its YEATS domain [13]. Moreover, the wildtype MLL protein is a member of a large multiprotein complex that contains many members of the human TFIID and SWI/SNF transcription complexes. Similar to MLL, Anc1 is a member of yeast TFIID and SWI/SNF complexes, and is thus intriguingly similar to MLL itself [14]. This, along with the fact that Anc1 and several of *MLL*'s leukemogenic fusion partners share the YEATS domain makes Anc1 a particularly interesting candidate for mechanistic analysis.

During DNA replication, template nucleotides that have been chemically modified or lack a base altogether frequently block advancement of the replication fork, and can even cause fork collapse. Unless a stalled replication fork is enabled to restart, the cell cannot properly complete DNA replication, resulting in cell cycle arrest and cell death. The post-replication repair (PRR) pathway, exemplified by the *RAD6* epistasis group in *S. cerevisiae*, employs a variety of mechanisms for restarting stalled replication forks. It is the least well characterized of the DNA repair pathways, and is generally divided into error-prone and error-free branches, although there is some disagreement as to the number and sub-branches therein [15]–[18]. The error prone branch employs specialized translesion DNA polymerases (i.e. Rev1, Pol ζ, Pol η) that individually, or in collaboration, allow replication past and beyond replication-blocking DNA lesions, usually in an error-prone manner. Such DNA lesion bypass enables continued replication, albeit at the cost of increased mutation, and renders the lesion available for subsequent DNA repair [19]. The error-free branch of PRR, still largely uncharacterized, competes with Rad52-mediated homologous recombination for substrates, and likely repairs these substrates by recombination between sister-strands, through either template strand switching or copy choice mechanisms [20]. The error-free branch of PRR is associated with a subset of the Rad6 epistasis group, including Rad6, Rad18, Srs2, Rad5, Ubc13 and Mms2 [18].

Rad6, an E2 ubiquitin conjugating enzyme, forms a heterodimer with Rad18, a ubiquitin ligase and single-strand DNA-dependent ATPase. Under appropriate conditions the Rad6/Rad18 heterodimer monoubiquitinates PCNA at lysine 164. PCNA thus modified activates the error-prone PRR pathway by recruiting translesion polymerases to the replication fork [21], [22]. Alternately, monoubiquitinated PCNA can serve as a substrate for polyubiquitination by the Rad5/Mms2/Ubc13 complex, leading to activation of the error-free pathway instead [21], [22]. Like Rad18, Rad5 is a single-strand DNA-dependent ATPase. Rad5 appears to play a complex role in these pathways, with evidence for its participation in error-prone translesion synthesis, and at least one putative branch of the error-free pathway, although its primary role is considered to be in the error-free branch [15], [17], [18], [23], [24]. It has recently been shown to have a specialized helicase activity for replication fork regression that may be important for template switching [25]. Srs2 (“*S*uppressor of *R*ad*6*”), a DNA-dependent ATPase and helicase, strips Rad51 from single-stranded DNA, preventing Rad51 from sequestering the DNA for homologous recombination, and allowing PRR pathway members to access the substrate, directing to toward synthesis-dependent strand annealing instead [26], [27]. Thus, Srs2 acts as the gatekeeper to all of postreplicative repair, although it only suppresses damage-induced sensitivity and mutagenesis in mutants of the error-free branch of PRR [28].

In this study we investigate the basis of *anc1*Δ's sensitivity to alkylating agents. We show that *ANC1* defines a new branch in the PRR pathway, one that is error-free, promotes cell survival in the presence of DNA damaging agents, and suppresses both induced and spontaneous mutation, including the expansion of CAG triplet repeats.

## Results

### Analysis of Anc1-containing complexes

As discussed earlier, Anc1 is a member of several RNA Pol II-related multi-protein complexes, namely TFIID, TFIIF, RSC, SWI/SNF, INO80, NuA3 and Mediator. Given these associations, we set out to determine whether the role of Anc1 in providing alkylation resistance could be assigned to one or more of these complexes, bearing in mind that Anc1 might provide resistance independently of these complexes. We therefore checked the sensitivity of mutants deleted for the non-essential members for each complex. We reasoned that if deletion mutants for other members of a particular protein complex share *anc1*Δ's damage sensitivity profile, this would pinpoint the complex via which Anc1 helps cells survive after chemical damage.

Using data from our genome-wide DNA damage sensitivity phenotyping screen, the non-essential members of Anc1's constituent complexes were checked for MMS, 4NQO and UV sensitivity [1], [2] (Figure S1). For two of the seven Anc1-containing complexes, namely TFIID and TFIIF, Anc1 is the only non-essential member, so these complexes could not be interrogated. Of the five complexes containing non-essential members in addition to Anc1, four have a majority of subunits that, when deleted, share *anc1*Δ's sensitivity to MMS, 4NQO or UV; these are Mediator, SWI/SNF, INO80, and RSC excluding only NuA3 (Figure S1). The damage sensitivity of strains deleted for several subunits in four out of five complexes demonstrates that the role of Anc1 in survival after DNA damage is likely to be tied to the functions of at least four of its protein complexes.

### Alkylation damage induces cell cycle arrest in Anc1 deficient cells

**Figure 1:**
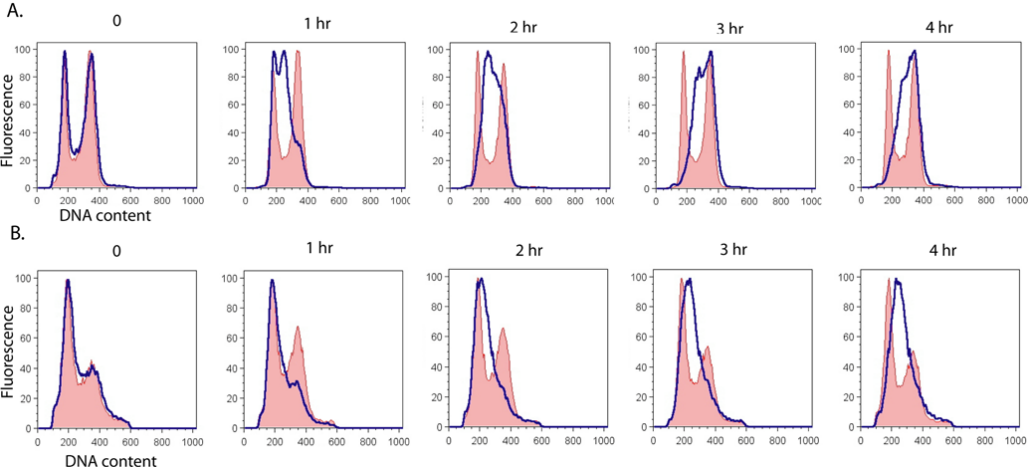
Cell cycle distribution of wildtype and *anc1*Δ asynchronous cells before and after MMS exposure. A. WT cells, B. *anc1*Δ cells. When log-phase growing cells in YPD reached an OD(600) of 0.2, MMS was added to half of the cells at a concentration of 0.015%, and aliquots were removed at the indicated times to monitor cell cycle distribution by flow cytometry. Profiles of untreated cells are shown in red shading, and profiles of treated cells are shown with a blue trace.

Many cell cycle-related genes are critical for survival after alkylation damage; indeed, ∼45% of known cell cycle regulation genes were found to be MMS sensitive in our genome-wide screen for genes involved in damage resistance [2]. Strains mutated in genes that are necessary for the Mec1-mediated DNA damage checkpoint (i.e. *MEC1*, *RAD53, RAD9, RAD17, RAD24*) are more sensitive to killing by MMS than wildtype strains [29], [30]. Given the sensitivity of the *anc1*Δ strain to MMS and 4NQO damage, it seemed plausible that their sensitivity may be due their failure to arrest in response to DNA damage [31]. To assess the effect of Anc1 on the Mec1-mediated DNA damage checkpoint, we analyzed cell cycle progression in wild-type and *anc1*Δ yeast cultures in the presence of MMS [30] (Figure 1).

**Figure 2:**
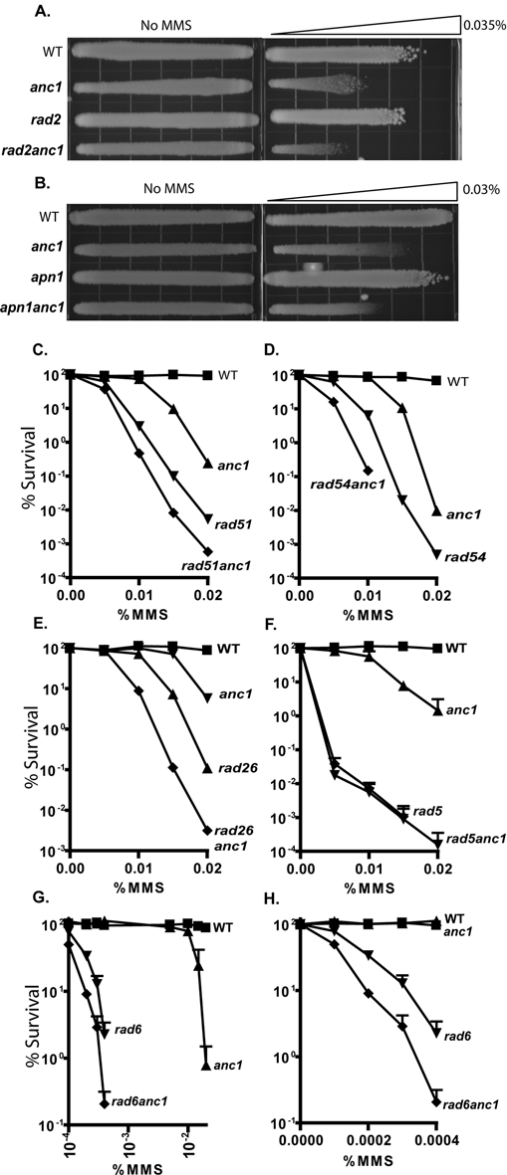
Epistasis analysis of *ANC1* in known DNA repair pathways. Survival after chronic MMS treatment for: A. YPD gradient plate with maximum dose 0.03% MMS, B. YPD gradient plate with maximum dose 0.035% MMS, C. WT (▪), *anc1*Δ (▴), *rad51*Δ (▾), *rad51anc1*Δ (♦). D. WT (▪), *anc1*Δ (▴), *rad54*Δ (▾), *rad54anc1*Δ (♦), E. WT (▪), *anc1*Δ (▴), *rad26*Δ (▾), *rad26anc1*Δ (♦), F. WT (▪), *anc1*Δ (▴), *rad5*Δ (▾), *rad5anc1*Δ (♦). Log-phase cells were diluted and plated on freshly poured MMS-containing YPD-agar plates or onto control plates with no MMS. Colonies were allowed to grow at 30°C for 2–5 days before counting. At least two replicates were counted per trial. Serial dilution and gradient plate replicates available in Figure S2.

**Figure 3:**
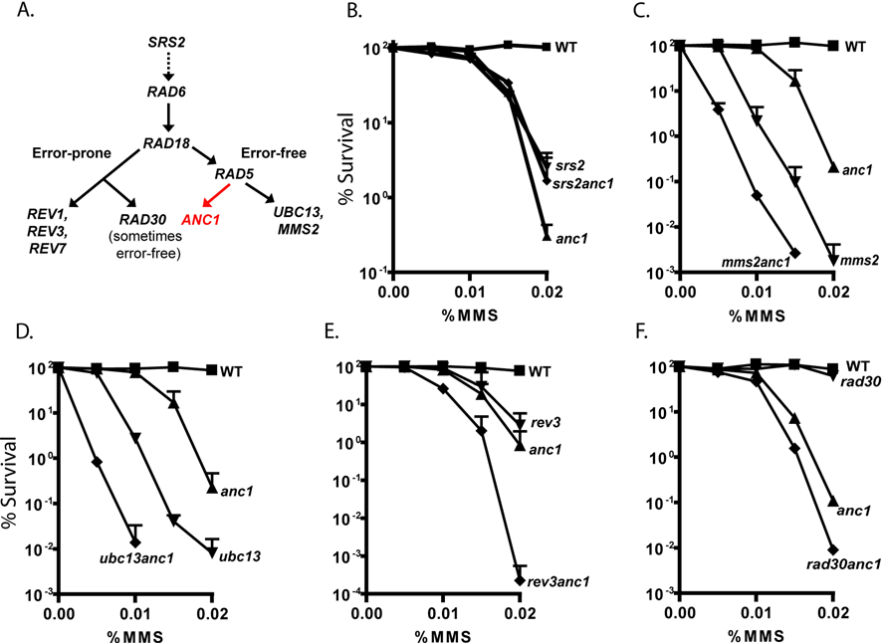
Epistasis analysis of *ANC1* with PRR pathway members. A. Genetic relationships within the PRR pathway in yeast. Epistasis was determined by sensitivity of mutants to damaging agents. *srs2*Δ only suppresses the damage sensitivity of *rad5*Δ, *ubc13*Δ and *mms2*Δ mutants (modified from Ulrich, 2006). Survival after chronic MMS treatment for: B. WT (▪), *anc1*Δ (▴), *srs2*Δ (▾), *srs2anc1*Δ (♦), C. WT (▪), *anc1*Δ (▴), *mms2*Δ (▾), *mms2anc1*Δ (♦) D. WT (▪), *anc1*Δ (▴), *ubc13*Δ (▾), *ubc13anc1*Δ (♦), E. WT (▪), *anc1*Δ (▴), *rev3*Δ (▾), *rev3anc1*Δ (♦) F. WT (▪), *anc1*Δ (▴), *rad30*Δ (▾), *rad30anc1*Δ (♦). We made several attempts to create a *rad18anc1*Δ strain for epistasis analysis, but were unable to produce the double mutant by either mating or recombination, even in the presence of a covering plasmid bearing an intact *RAD18*. Log-phase cells were diluted and plated on freshly poured MMS-containing YPD-agar plates or onto control plates with no MMS. Colonies were allowed to grow at 30°C for 2–5 days before counting. Results are average of at least 2 replicates, error bars = SD, except in F.; gradient plate replica for F. in Figure S2. We were unable to create a *rad18anc1*Δ double mutant by either mating or transformation, even with Rad18 expressed from a covering plasmid during transformation.

**Figure 4:**
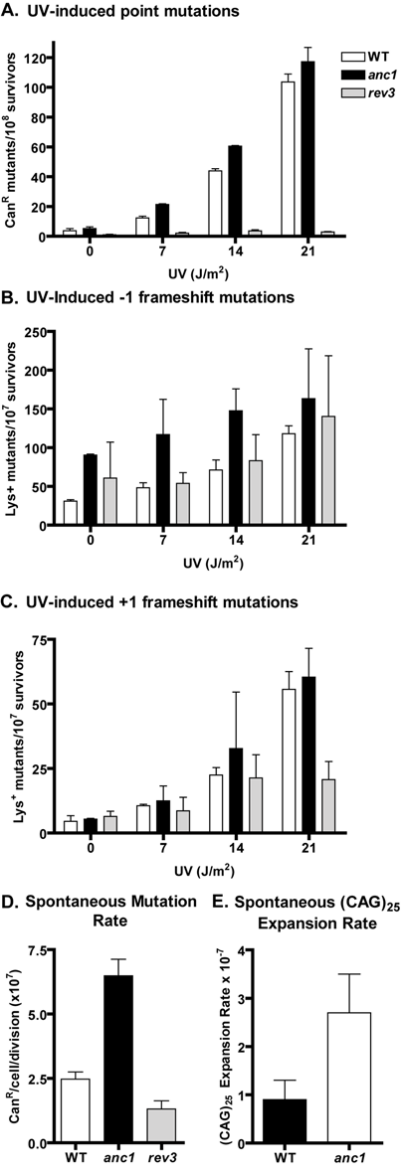
UV-induced point and frameshift mutagenesis and spontaneous mutagenesis. A. Induced point mutagenesis in BY4741 background: wildtype, *anc1*Δ and *rev3*Δ cells were exposed to UV doses as indicated. Results are mean of two replicates, +/−SD. B. and C. Induced frameshift mutagenesis in CG379-A_12_ and CG379-A_14_ backgrounds: WT and *anc1*Δ frameshift reversions to a functional Lys2 gene. Cells were exposed to UV doses as indicated. Results are mean of two replicates, +/−SD. D. Spontaneous point mutagenesis, +/−SD, measured as described in Materials and Methods. E. Spontaneous expansion in (CAG)_25_ repeats, +/−SD, measured as described in Materials and Methods.

As previously shown, a moderately toxic dose of MMS (0.015%) induced a Mec1-dependent S-phase arrest in wildtype *S. cerevisiae* [30]. The 0.015% dose of MMS used in this experiment causes minimal killing in wildtype and only moderate killing in *anc1*Δ strains (Figures 2, 3 and 4). Although *anc1*Δ strains grow more slowly than wildtype [32], the MMS-induced S-phase arrest is clearly observed in both the wildtype and *anc1*Δ strains (Figure 1); it is important to note that no such arrest is observed in *mec1*Δ *-1*, *rad53*Δ, *rad9*Δ, *rad17*Δ and *rad24*Δ [29], [30]. Interestingly, even when arrest is induced in several of these deletion strains, it has been found that they do not undergo normal repair [33], [34]. However, *anc1*Δ cells take longer than wildtype to reach an arrested state, and also take longer to move through S phase (Figure 1). This lag may be a result of the following: (i) *anc1*Δ's slow growth rate; (ii) a slower release from the checkpoint; or (iii) a more strongly induced cell cycle arrest (Figure 1). Comparing the untreated cell cycle profiles of *anc1*Δ and wildtype, we observed that *anc1*Δ cells spend much longer in G1 than do wildtype cells, presumably contributing to their slow growth phenotype (Figure 1). The reason for a prolonged G1 in *anc1*Δ cells is unclear, but may be linked to a role for Anc1 either in leaving G1 or in starting S phase. What does seem clear is that the sensitivity of *anc1*Δ cells is not due to a complete failure to arrest at the Mec1-mediated DNA damage checkpoint, although there does seem to be a delay in triggering this S-phase checkpoint [29].

### Epistasis of ANC1 with established DNA Repair pathways

To determine if *ANC1* functions within a canonical DNA repair pathway, we examined whether *anc1*Δ could be assigned to established DNA repair epistasis groups, namely nucleotide excision repair (here represented by *rad2*Δ), base excision repair (*apn1*Δ), homologous recombination (*rad51*Δ, *rad54*Δ), transcription coupled repair (*rad26*Δ) or postreplicative repair (PRR) (*rad5*Δ and *rad6*Δ) (Figure 2, Figure S2). The MMS sensitivity of double mutant strains was compared to each of the single mutants as well as wildtype yeast. With the exception of *rad5anc1*Δ, the double mutants all showed additive or synergistic effects compared to the single mutants (Figure 2, Figure S2). The sensitivity of the *rad5anc1*Δ double mutant matches that of the *rad5*Δ single mutant, indicating that *ANC1* shares a genetic pathway with *RAD5*, a member of the postreplicative repair pathway (Figure 2F). *rad6*Δ strains are extremely sensitive to MMS, roughly 100X more sensitive than *anc1*Δ, so we used two sets of MMS doses to observe sensitivity in this epistasis test (Figures 3G and H). At the MMS doses to which *anc1*Δ cells begin to show sensitivity, the sensitivity of the *rad6*Δ and the *rad6anc1*Δ strains was so great that survival could not be measured. But, looking at MMS doses on a log scale, we observed an possibly epistatic relationship between *ANC1* and *RAD6* (Figure 2G). A closer examination of the extremely low MMS dose range where the survival of *rad6*Δ and *rad6anc1*Δ strains can be accurately measured may, however, reveal a synergistic relationship between these two genes (Figure 2H).

*RAD5* is known to belong to the error-free branch of PRR, although studies have shown an additional role for Rad5 in the error-prone branch of the pathway [15], [23], [35]. *RAD5* has a complex relationship with other members of the error-free branch of PRR: the *rad5mms2*Δ double mutant has an additive effects for UV or MMS induced cytotoxicity compared to the single mutants [16], and Mms2/Ubc13-dependent and -independent roles for Rad5 have recently been described [15]. *RAD6*, on the other hand, operates upstream of the branching between the error-prone and error-free pathways (Figure 3A).

After establishing *ANC1's* genetic relationship with *RAD5* and *RAD6*, we assayed the epistasis between *ANC1* and other members of the PRR pathway, namely *SRS2*, *MMS2*, *UBC13*, *REV3*, and *RAD30* (Figure 3). The genetic relationships between the genes in the PRR pathway and the number of branches therein are a subject of some disagreement, but the pathway is generally divided into error-prone and error-free branches [15]–[18] (Figure 3A). Like *RAD5*, *SRS2* was also found to be in the same genetic pathway as *ANC1*, with the *srs2*Δ mutation suppressing the MMS sensitivity of *anc1*Δ (Figure 3B). The suppression of *anc1*Δ sensitivity in the *srs2anc1*Δ double mutant is consistent with earlier observations that the *srs2*Δ deletion suppresses the MMS sensitivity of several mutants in the error-free branch of the postreplicative repair pathway, including *rad5*Δ, *ubc13*Δ and *mms2*Δ [28], [36]. These data support the hypothesis that Anc1 functions in the error-free branch of postreplicative repair, downstream of Srs2.

Epistasis analysis of *ANC1* with *RAD18* was not carried out because we were unable to create a *rad18anc1*Δ double mutant by either mating or transformation, even with Rad18 expressed from a covering plasmid during transformation. The defective alpha-factor response and sporulation of *anc1*Δ have been previously noted [32]. Mutants for two other error-free pathway components, *MMS2* and *UBC13*, showed a synergistic pattern of sensitivity to MMS when combined with the *ANC1* mutation (Figure 3C, D). From this we infer that Anc1 might act on the same type of damage as Mms2/Ubc13, but through a different pathway. The epistasis of *ANC1* with *RAD5* does not help us determine to which branch of PRR it belongs, as *RAD5* has a role in the error-prone as well as the error-free pathway. *SRS2, MMS2* and *UBC13* are all characterized members of the error-free branch, and given the epistasis of *ANC1* with *SRS2*, but synergistic relationship with *MMS2* and *UBC13*, the role of *ANC1* in PRR does not fit into the canonical error-free branch.

Because *ANC1* is synergistic rather than epistatic with the *MMS2* and *UBC13* members of the error-free branch of the PRR pathway, we determined whether *ANC1* lies in the error-prone pathway [37]. We analyzed the alkylation sensitivity of *anc1*Δ in combination with *rev3*Δ or *rad30*Δ, mutants in two translesion DNA polymerases involved in PRR: *REV3* (with *REV7*) encodes DNA polymerase ζ, an error-prone polymerase, and *RAD30* encodes polymerase η, a polymerase that is sometimes characterized as error-prone, and sometimes as error-free depending on the type of lesion being bypassed [38]. The *rev3anc1*Δ double mutant showed an additive MMS-sensitivity phenotype relative to the single mutants, indicating that Anc1 probably lies in a non-overlapping pathway with Rev3 (Figure 3E). The *rad30anc1*Δ double mutant, however, appeared to have synergistic sensitivity when compared to the sensitivities of the single mutants, possibly indicating a partially overlapping function between Rad30 (Pol η) and Anc1 (Figure 3F). Thus, with respect to its genetic pathway, *ANC1* appears to be independent from both the error-prone and error-free branches of postreplicative repair. Taken together, from the lack of epistasis between *ANC1* and error-free branch members *UBC13* and *MMS2*, and from the lack of epistasis with error-prone branch members *REV3* and *RAD30*, we infer that *ANC1* functions in a genetic pathway that is independent from the two established branches, and therefore, may define a new branch of PRR (Figure 3A).

Given Anc1's presence in several transcriptionally-important complexes (Figure S1), it seems possible that Anc1's interaction with the genes of the postreplicative repair pathway may take place on a transcriptional level. To determine whether Anc1 has an effect on the transcription of genes involved in PRR, we isolated total RNA from wildtype and *anc1Δ* cells, and assayed the transcriptional levels of the PRR genes using Affymetrix microarrays (Table 1). This analysis demonstrated that none of the PRR pathway members had significant changes in expression between the wildtype and *anc1Δ* strains, but that, as expected, the expression level of *ANC1* itself was considerably and significantly lower in the *anc1Δ* strain than in wildtype (Table 1).

**Table 1:** Difference in gene expression between PRR members and *ANC1.*

Genes of Interest	Representative Public ID	WT/*anc1* Fold Change	Adjusted p-value
*ANC1*	YPL129W	28.63	0.00*
*RAD6*	YGL058W	1.25	0.08
*RAD18*	YCR066W	1.00	0.76
*RAD5*	YLR032W	1.24	0.39
*UBC13*	YDR092W	1.02	0.97
*MMS2*	YGL087C	1.34	0.18
*SRS2*	YJL092W	1.34	0.16
*REV1*	YOR346W	1.05	0.95
*REV3*	YPL167C	1.27	0.37

* p-value is significant.

### Induced and spontaneous mutagenesis in *anc1*Δ cells

As discussed, PRR has been divided into “error-prone” and “error-free” branches. When the error-prone pathway is impaired, cells become refractory to spontaneous and damage-induced mutagenesis; when the error-free pathway is impaired, cells become, if anything, more susceptible to damage-induced mutagenesis. Given that *ANC1* is not epistatic with all members of the error-free branch of postreplicative repair, it was important to determine whether *ANC1* acts in an error-free or error-prone pathway with respect to mutagenesis.

Yeast lacking Anc1 were assayed for both frameshift and point mutations as previously described [22], [39]–[41]. Disruptions of the *CAN1* gene, as monitored by canavanine resistance, detected predominantly point mutations (although deletions, duplications and gross chromosomal rearrangements can also disrupt *CAN1*) [40] Frameshift mutations were monitored by reversion of the *lys2* A_12_ and A_14_ alleles containing mononucleotide runs of adenines [39]. Functional *LYS2* is only expressed after a −1 or +1 frameshift mutation in *lys2* A_12_ and *lys2* A_14_, respectively [39]. Rev3 is a well-characterized member of the error-prone branch of PRR, and is used here as a positive control for monitoring the phenotype associated with a deficiency in an error-prone pathway (Figure 4).

*ANC1* deleted cells were slightly more sensitive than wildtype yeast to UV-induced point mutations and −1 framshift mutations; in contrast, UV-induced +1 frameshift mutations were similar between *anc1*Δ and wildtype. (Figure 4A, B, C). At the *CAN1* locus *rad5*Δ has been observed to result in a slight increase in UV-induced point mutagenesis compared to wildtype, although the induced mutagenesis in *rad5*Δ strains has been previously characterized as being very dependent on the mutational target being assayed [15], [42], [43]. This is consistent with the slight increase in induced mutagenesis observed in our *anc1*Δ strain at the *CAN1* locus (Figure 4A). In contrast, the *rev3*Δ deleted strain is refractory to UV-induced point mutations compared to both wildtype and *anc1*Δ strains (Figure 4A). Thus, *anc1*Δ's sensitivity to damage-induced mutagenesis is consistent with Anc1 acting in an error-free rather than an error-prone pathway.

Previous studies have shown an increase in spontaneous mutation rates among mutants in the error-free branch of PRR, and a decrease in the spontaneous mutation rate among mutants in the error-prone branch [17], [37], [44]. Here we show that the deletion of *ANC1* results in an increased frequency of spontaneous -1 frameshift mutations (Figure 4B), and also in an increased spontaneous base pair substitution mutation rate compared to wildtype (Figure 4D). Note that the *rev3*Δ control displays a decreased spontaneous base pair substitution mutation rate compared to wildtype. Thus, in terms of both induced and spontaneous mutation, the newly defined Anc1 branch of PRR is clearly error-free, protecting *S. cerevisiae* from both cytotoxicity and mutagenesis.

### Anc1 protects against trinucleotide repeat expansions

It was recently reported that gene deletion for several members of the error-free branch of the PRR pathway, including *RAD5* and *SRS2*, results in an expansion of CAG and CTG trinucleotide repeats (TNRs); expansion of such repeats have been associated with Huntington's disease and myotonic dystrophy [45]–[47]. In those studies, it was observed that the disease-associated TNRs, but not dinucleotide repeats or non-disease associated TNRs, are prone to expansion, but not contraction, in cells deficient in the error-free branch of the PRR pathway [45].

To determine whether Anc1 plays a role in limiting CAG expansions like other members of the error-free PRR pathway, an *anc1*Δ deletion was introduced into a strain containing a (CAG)_25_ construct, and assayed for CAG expansions as described [45]. Like other PRR members, *anc1*Δ displays a statistically significant (three-fold) increase in CAG expansions compared to wildtype (Figure 4E). This expansion is statistically indistinguishable from those in *rad5*Δ and *mms2*Δ strains, although it is considerably lower than the expansion observed for several other PRR mutants [45]. These data indicate that Anc1, like other members of the error-free PRR pathway, plays a role in preventing the expansion of CAG trinucletide repeat sequences.

## Discussion

Anc1 is known to directly interact with the catalytic protein subunits for six of the seven Anc1-containing multi-protein complexes, including TFIID, TFIIF, RSC, Ino80, SWI/SNF and NuA3 9, 48, 49. Of the six subunits with which Anc1 directly interacts, Sth1, Ino80 and Snf2 are DNA-dependent ATPases/helicases with sequence similarity to the SNF2 family of DNA-dependent ATPases [9], [50], [51]. The other three catalytic subunits are Tsm1 and Tgf1 that are both involved in general transcription initiation, and Sas3, the catalytic subunit of NuA3 that acetylates histone H3 [9]. Given the interaction between Anc1 and the catalytic domains of nearly all of its component complexes, plus the putative interaction between histones and the Anc1 YEATS domain, it seems likely that Anc1 acts as a regulatory adapter between chromatin and the complexes that act upon it [9], [13]. The damage sensitivity of cells mutant in individual components of so many of these complexes suggests that Anc1 is involved in regulating transcription, chromatin remodeling, and as reported here, PRR, upon exposure to DNA damaging agents.

The *ANC1* transcript belongs to a minority of yeast transcripts that contain a splice site. It was recently reported that *ANC1* mRNA splicing is regulated by Cdc40, a protein involved in controlling cell cycle progression [31]. In the absence of *CDC40*, cells arrest in G2/M, and the addition of intronless *ANC1* cDNA only partially mitigates this arrest [31], [52] indicating that Cdc40 may have other splicing targets in addition to the *ANC1* mRNA, or may have yet another function. The slow transition out of G1 that we observed in *anc1*Δ cells is also observed in *cdc40*Δ cells [52], [53], and like *anc1*Δ, *cdc40*Δ mutants are sensitive to a variety of DNA damaging agents, including hydroxyurea, MMS, 4NQO and UV [2], [53]. However, the sensitivity of *cdc40*Δ cells to MMS or HU is not suppressed when intron-less *ANC1* cDNA is expressed [53]. Of relevance to this study, a temperature sensitive allele of *cdc40*Δ was shown to be epistatic to an allele of *rad6*Δ in terms of MMS sensitivity during log phase growth, although neither allele was characterized as being null [54]. Since a correctly spliced *ANC1* transcript does not suppress the MMS or HU sensitivity of *cdc40*Δ cells, we must conclude that Cdc40 has another function in allowing cells to survive after DNA damage that is independent from *ANC1* transcript splicing. Like *ANC1*, *UBC13* and *MMS2* are intron-containing genes in the PRR pathway [55]. Given the observed epistasis between alleles of *cdc40*Δ and *rad6*Δ after MMS treatment [54], and the failure of the correctly spliced *ANC1* transcript to complement a *cdc40*Δ mutant's damage sensitivity [53], it is worth exploring whether Cdc40 mediates the splicing of the *MMS2* and/or *UBC13* transcripts as well.

Several pieces of evidence support a role for Anc1 in the PRR pathway. Based on the suppression of *anc1*Δ's sensitivity by *srs2*Δ, *ANC1* can be placed genetically downstream of *SRS2*, as was previously observed for other members of the error-free PRR pathway [28], [35], [36]. *ANC1* also shares a genetic pathway with *RAD5*, a downstream member of the error-free pathway and possibly with *RAD6*, which lies between *SRS2* and *RAD5* in the genetic model of the PRR pathway (Figure 3A). The synergism observed at low MMS doses between *rad6*Δ and *anc1*Δ may imply a role for Anc1 that is partially parallel to that of Rad6, possibly indicating that Anc1 is involved in one of the several functions of Rad6. The lack of epistasis between *ANC1* and other error-free branch members *MMS2* and *UBC13* provides evidence for a new, Mms2/Ubc13 independent branch of the PRR pathway. Given that we were unable to create a *rad18anc1*Δ double mutant by mating or transformation, even in the presence of a covering plasmid bearing an intact *RAD18*, we do not yet know whether Rad18 also plays a role in the new pathway defined by Anc1; however, since no Rad6-independent role for Rad18 has been described, it seems likely that Rad18 also plays a role in the Anc1-branch of PRR.

Two types of mutagenesis data indicated that the Anc1-containing branch of the PRR pathway deals with DNA damage in an error-free manner. First, the *ANC1* deletion, similar to deletions for most members of the error-free PRR pathway [28], [43], causes an increase in both induced and spontaneous point mutation compared to wildtype. Second, *anc1*Δ mutants display a significant increase in the expansion of CAG tri-nucleotide repeats compared with wildtype, a trait that was recently identified in all of the tested members of the error-free branch of the PRR pathway, including *srs2*Δ and *rad5*Δ [45]. These mutagenesis data are consistent with a role for Anc1 in error-free PRR.

The role of Anc1 in PRR may be crucial for understanding the interaction of key players in the cellular response to DNA damage. Anc1 interacts physically with Mus81, a structure-specific endonuclease in the XPF family involved in cleaving stalled replication forks [56], [57]. Mus81 forms a heterodimer with Mms4 for its endonuclease activity, and deletions of either partner results in sensitivity to MMS and 4NQO [1], [2]. Mus81 is speculated to be involved with the PRR pathway (in addition to its better characterized role in homologous recombination) by means of its cleavage of the stalled replication forks that the PRR pathway acts upon [57]. Furthermore, there is genetic evidence in *S. pombe* that *srs2*Δ and *mus81*Δ are epistatic with respect to their MMS, UV and HU sensitivities [58], although in *S. cerevisiae* the *mms4srs2*Δ double mutant displays a synergistic effect compared with either of the single mutants after MMS or UV treatment, suggesting that their pathways may partially overlap [59] Having demonstrated the membership of *ANC1* in the error-free branch of PRR, it seems likely that the physical interaction between Mus81 and Anc1 relates to Mus81's cleavage function in PRR. The method by which Mus81 recognizes its substrates is not well understood, but it seems possible that Anc1, through its presumed interaction with histones [13], allows the Mus81 endonuclease access to sites where its cleavage will initiate the sister-strand recombination that drives error-free PRR.

Given the direct interaction between the YEATS domain of ENL with histones H1 and H3, and the interaction of Anc1 with the catalytic subunits of so many transcriptionally-important complexes [9], [13], it may be hypothesized that Anc1 acts as a DNA-damage mediated adapter between chromatin, transcription and PRR repair at or near sites of DNA damage. Since transcription generally continues through S-phase, while DNA is being replicated, the collision of the transcriptional machinery and stalled replication forks is thought to be a common event [60]. In recent years there has been considerable interest in the phenomena of transcription-associated mutation (TAM) and transcription-associated recombination (TAR), which characterize the mutagenesis and recombination that occur when the transcription and replication machineries collide [60]. Mediation of the interaction between these machineries by a common member (Anc1) of the transcription complexes is a possibility worthy of further exploration. It is possible that the new branch of postreplicative repair represented by Anc1 is responsible for mediating the repair of replication forks that have stalled as a result of the collision between transcription and replication machineries. Furthermore, the role of the human YEATS containing leukemia-associated proteins, ENL, AF9 and GAS41, in both the human post-replication repair pathway, and in polyglutamine expansions such as those associated with Huntington's disease is certainly worthy of further exploration, and may provide insight into the molecular basis of such disparate diseases as leukemia and Huntington's disease.

## Materials and Methods

### Yeast Strains and Media

Yeast strains used in this study are listed in Table 2. Yeast strains were grown in standard media, including YPD and synthetic complete (SC) medium. All strains are congenic with the BY4741 background (MAT*a his*3Δ*1 leu2*Δ*0 met15*Δ*0 ura3*Δ*0*), except for the spontaneous mutagenesis and the trinucleotide repeat assays as specified below in Induced and Spontaneous Mutagenesis Assays and Table 2. Double mutants were created by transformation of an *anc1*Δ::URA3 linear cassette into G418 resistant strains from the genome-wide deletion collection (Invitrogen-ResGen) [61], [62]. Homologous ends allowed the cassette to recombine into the endogenous location of *ANC1* [61]. Constructs were confirmed by PCR and/or DNA sequencing.

**Table 2:** Strains used in this study.

Strain	Genotype	Reference
*S. cerevisiae*		
BY4741	*MATa his3Δ1 leu2Δ0 met15Δ0 ura3Δ0*	Brachmann et al. 1998
BY4741anc1	BY4741 *anc1Δ::URA3*	this study
BY4741rad2	BY4741 *rad2Δ::kanMX4*	Brachmann et al. 1998
BY4741rad2anc1	BY4741 *rad2Δ::kanMX4 anc1Δ::URA3*	this study
BY4741apn1	BY4741 *apn1Δ::kanMX4*	Brachmann et al. 1998
BY4741apn1anc1	BY4741 *apn1Δ::kanMX4 anc1Δ::URA3*	this study
BY4741rad51	BY4741 *rad51Δ::kanMX4*	Brachmann et al. 1998
BY4741rad51anc1	BY4741 *rad51Δ::kanMX4 anc1Δ::URA3*	this study
BY4741rad54	BY4741 *rad54Δ::kanMX4*	Brachmann et al. 1998
BY4741rad54anc1	BY4741 *rad54Δ::kanMX4 anc1Δ::URA3*	this study
BY4741rad26	BY4741 *rad26Δ::kanMX4*	Brachmann et al. 1998
BY4741rad26anc1	BY4741 *rad26Δ::kanMX4 anc1Δ::URA3*	this study
BY4741rad5	BY4741 *rad5:Δ:kanMX4*	Brachmann et al. 1998
BY4741rad5anc1	BY4741 *rad5Δ::kanMX4 anc1Δ::URA3*	this study
BY4741srs2	BY4741 *srs2Δ::kanMX4*	Brachmann et al. 1998
BY4741srs2anc1	BY4741 *srs2Δ::kanMX4 anc1Δ::URA3*	this study
BY4741rad6	BY4741 *rad6Δ::kanMX4*	this study
BY4741rad6anc1	BY4741 *rad6Δ::kanMX4 anc1Δ::URA3*	this study
BY4741mms2	BY4741 *mms2Δ::kanMX4*	Brachmann et al. 1998
BY4741mms2anc1	BY4741 *mms2Δ::kanMX4 anc1Δ::URA3*	this study
BY4741ubc13	BY4741 *ubc13Δ::kanMX4*	Brachmann et al. 1998
BY4741ubc13anc1	BY4741 *ubc13Δ::kanMX4 anc1Δ::URA3*	this study
BY4741rev3	BY4741 *rev3Δ::kanMX4*	Brachmann et al. 1998
BY4741rev3anc1	BY4741 *rev3Δ::kanMX4 anc1Δ::URA3*	this study
BY4741rad30	BY4741 *rad30Δ::kanMX4*	Brachmann et al. 1998
BY4741rad30anc1	BY4741 *rad30Δ::kanMX4 anc1Δ::URA3*	this study
CG379-A12	*MATα ade5Δ1 his7Δ2 leu2Δ3, 112 trp1Δ289 ura3Δ52 lys2::InsE-A12*	Tran et al., 1997
CG379-A14	*MATα ade5Δ1 his7Δ2 leu2Δ3, 112 trp1Δ289 ura3Δ52 lys2::InsE-A14*	Tran et al., 1997
CG379-A12anc1	CG379-A12 *anc1Δ::kanMX4*	this study
CG379-A14anc1	CG379-A14 *anc1Δ::kanMX4*	this study
CG379-A12rev3	CG379-A12 *rev3Δ::HIS3*	Rusyn et al. (in preparation)
CG379-A14rev3	CG379-A14 *rev3Δ::kanMX4*	Klapacz et al (in preparation)
BL035	*MATα trp1Δ ura3Δ52 ade2Δ ade8Δ hom3Δ10 his3Δ kpn1Δ met4Δ met13Δ leu2Δ*	Daee et al. 2007
BL035anc1	BL035 *anc1Δ::kanMX4*	this study

### Flow Cytometry

Log phase cells were washed twice in 50 mM Tris pH 7.8, resuspended in 50mM Tris pH 7.8 containing RNase A (1 mg/ml) and incubated at 37°C overnight. Cells were pelleted and resuspended in 55 mM HCl containing 5 mg/ml Proteinase K, incubated at 37°C for 30 min, washed once with 200mM Tris pH 7.5, 211 mM NaCl, 78mM MgCl_2_, then resuspended in the same buffer with 1mg/ml of propidium iodide before assaying by flow cytometry using a FACScan cytometer (Becton Dickinson) and CellQuest Pro software. Two independent assays were performed to confirm reproducibility, and analysis was performed using FlowJo software Version 6.4.7.

### Sensitivity of deletion strains to DNA damaging agents in the genome-wide screen

The sensitivity of every non-essential gene deletant in *S. cerevisiae* was previously determined [1], [2]. Relative sensitivity values were generated using a scoring scheme that allocated values of 4, 3, 2, or 1 depending on the concentration of agent where strain sensitivity was identified; 4 is allocated to the lowest, and 1 is allocated to the highest concentration of damaging agent in the plate. These values were allowed to accumulate in each replicate, and then they were summed across all replicates. For example, in replicate 1, strains sensitive to all concentrations of agents received a score of 10 (4+3+2+1), and this was summed over all 3 replicates for a final score of 30 (10+10+10). Damage-sensitive strains had scores that ranged from 30 (most sensitive) to 2 (least sensitive) [2]. All data is available at http://genomicphenotyping.mit.edu/newpages/source2.html.

### RNA Extraction

Three independent colonies of both wildtype and *anc1*::G418^R^ were grown overnight, then diluted and grown into log phase for 4–5 hours in YPD. RNA was extracted using Qiagen's RNeasy Mini Kit, checked for quality using an AgilentBioanalyzer and 20 ug of total RNA were hybridized using Affymetrix eukaryotic labeling protocols on Affymetrix YG-S98 microarrays (Cogenics, North Carolina).

### Analysis of Microarray Data

Repair proficient and deficient strains were analyzed in triplicate on YG-S98 arrays. Normalization was carried out using the Robust Multichip Average (RMA) algorithm [63]. Arrays were analyzed using Microarray Suite 5.0 to obtain Absent/Present calls and filtered for transcripts that were not expressed in any experiment. Differential gene expression was calculated using a dual filtering criteria; (1) an estimation of statistical significance through the Local Pooled Error test (LPE) [64] calculated using S-Plus Array Analyzer with an adjustment for false discovery rate calculation of p value of <0.05 (Benjamini Hochberg) and (2) a fold change (FC) limit of 1.5. The raw data files from this experiment have been submitted to the Gene Expression Omnibus Database (www.ncbi.nlm.nih.gov/geo), accession number GSE12150.

### Survival Curves/Epistasis Assays

Log-phase cells were diluted and plated on freshly poured MMS-containing YPD-agar plates or onto control plates with no MMS. Colonies were allowed to grow at 30°C for 2–5 days, depending on rate of growth for each strain, and survival was calculated by dividing the number of surviving colonies at a given MMS dose by the number of colonies that grew in the untreated sample. At least two replicates were counted per trial.

### Induced and Spontaneous Mutagenesis Assays

Yeast strains CG379-A_12_ and CG379-A_14_ from [39] revert by −1 and +1 frameshifts in *LYS2::InsE-A_12_ and LYS2::InsE-A_14_*, respectively, were used to measure frameshift mutation frequencies. These strains are isogenic with CG379 (*MAT*α *ade5*Δ*1 his7*Δ*2 leu2*Δ*3, 112 trp*Δ*289 ura*Δ*52*) [39]. Frameshifts were calculated by comparing the number of Lys^+^ revertants growing on Lys^−^ media to the number of colonies on a YPD control. Point mutation frequencies were measured in a BY4741 background. Canavanine-resistant mutations were measured on synthetic complete medium containing 0.004% (or 30 mg/liter) canavanine [40]. In the induced mutagenesis assay, UV doses of 0, 7, 14 and 21 J/m^2^ were administered using a UV Stratalinker 2400 (Stratagene). Cells were grown into log phase, then serially diluted and plated onto YPD or Canavanine containing plates before exposure to UV. Colony formation on YPD was used to calculate the total number of cells plated on canavanine-containing plates, for a final calculation of mutants per 10^7^ survivors.

In the spontaneous mutagenesis assay, forward mutations at *CAN1* were determined based on the protocol previously described in Glassner et al. [41]. Briefly, an overnight culture of each strain was diluted to 4000 cells/ml in 5mL of YPD in 10 cultures. The cultures were allowed to grow at 30°C for 5 days, then a small amount diluted 10^5−^fold on YPD to assay for viable cells, and the remainder concentrated to 1 mL, and 100ul plated on 0.04% Canavanine-containing synthetic complete medium to assay for Can^R^ mutants. Mutagenesis rates were calculated using the Drake Formula [65].

### Trinucleotide Repeat Assay

Expansion rates were measured by fluctuation analysis as described previously [45], [66]–[68]. All experiments were conducted in BL035, a *leu2* version of the wild type strain MW3317-21A (*MATα trp1*Δ *ura3*Δ*52 ade2*Δ *ade8*Δ *hom3*Δ*10 his3*Δ *kpn1*Δ *met4*Δ *met13*Δ) [69]. (CAG)_25_ tracts were cloned into a yeast promoter-reporter construct that allows spacing-sensitive expression of the downstream *URA3* reporter. Yeast cells harboring an expansion of four or more repeats do not express *URA3* and are identified by their resistance to 5-fluoroorotic acid. Mutation rates are calculated by the method of the median [70]. Six independent clones were tested to ensure reproducibility. Single colony PCR analysis of expansions were done as previously described and rates were corrected by multiplying the percent *bona fide* expansions by the apparent mutation rates obtained by fluctuation analysis [68]. All statistical analyses were performed using the T-test (two-tailed distribution and two-sample equal variance) in Microsoft Excel and P-values less than 0.05 were considered statistically significant.

## Supporting Information

Figure S1 Sensitivity of strains deleted for non-essential members of Anc1-containing complexes to MMS and 4NQO and UV. Values for increasing sensitivities from 2–30 were calculated as described in Materials and Methods, by Begley et al, 2004 and as displayed at http://genomicphenotyping.mit.edu/newpages/source2.html. A) Mediator complex, B) SWI/SNF complex, C) Ino80 complex, D) RSC complex-although RSC14 is not essential, there is no sensitivity data available, E) NuA3 complex.
(16.78 MB DOC)

Figure S2 Sensitivity of DNA repair pathway members. A. Five-fold serial dilutions on YPD containing 0.01% MMS. Cell concentrations were normalized after overnight growth. B., C., D. Gradient plates on YPD containing the indicated concentrations of MMS. Cell concentrations were normalized after overnight growth.
(23.67 MB DOC)
