# Supplementary figures and images for: Anc1, a Protein Associated with Multiple Transcription Complexes, Is Involved in Postreplication Repair Pathway in S. cerevisiae
Source: PLoS One. 2008 Nov 13;3(11):e3717. doi: 10.1371/journal.pone.0003717 (PMC2579579; doi:10.1371/journal.pone.0003717)

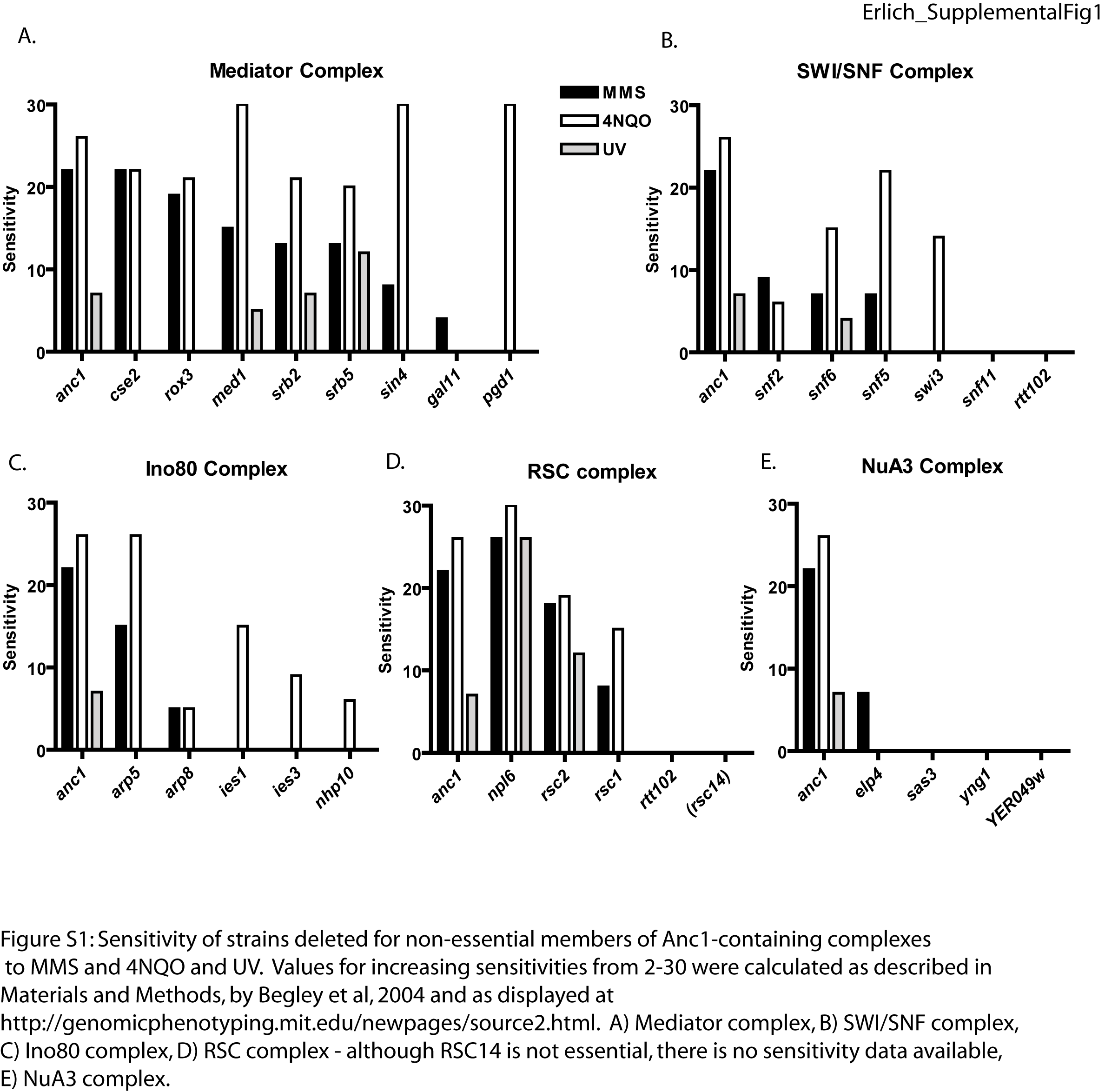

Supplement: Figure S1 — Sensitivity of strains deleted for non-essential members of Anc1-containing complexes to MMS and 4NQO and UV. Values for increasing sensitivities from 2–30 were calculated as described in Materials and Methods, by Begley et al, 2004 and as displayed at http://genomicphenotyping.mit.edu/newpages/source2.html. A) Mediator complex, B) SWI/SNF complex, C) Ino80 complex, D) RSC complex-although RSC14 is not essential, there is no sensitivity data available, E) NuA3 complex. (16.78 MB DOC) [file pone.0003717.s001.doc]

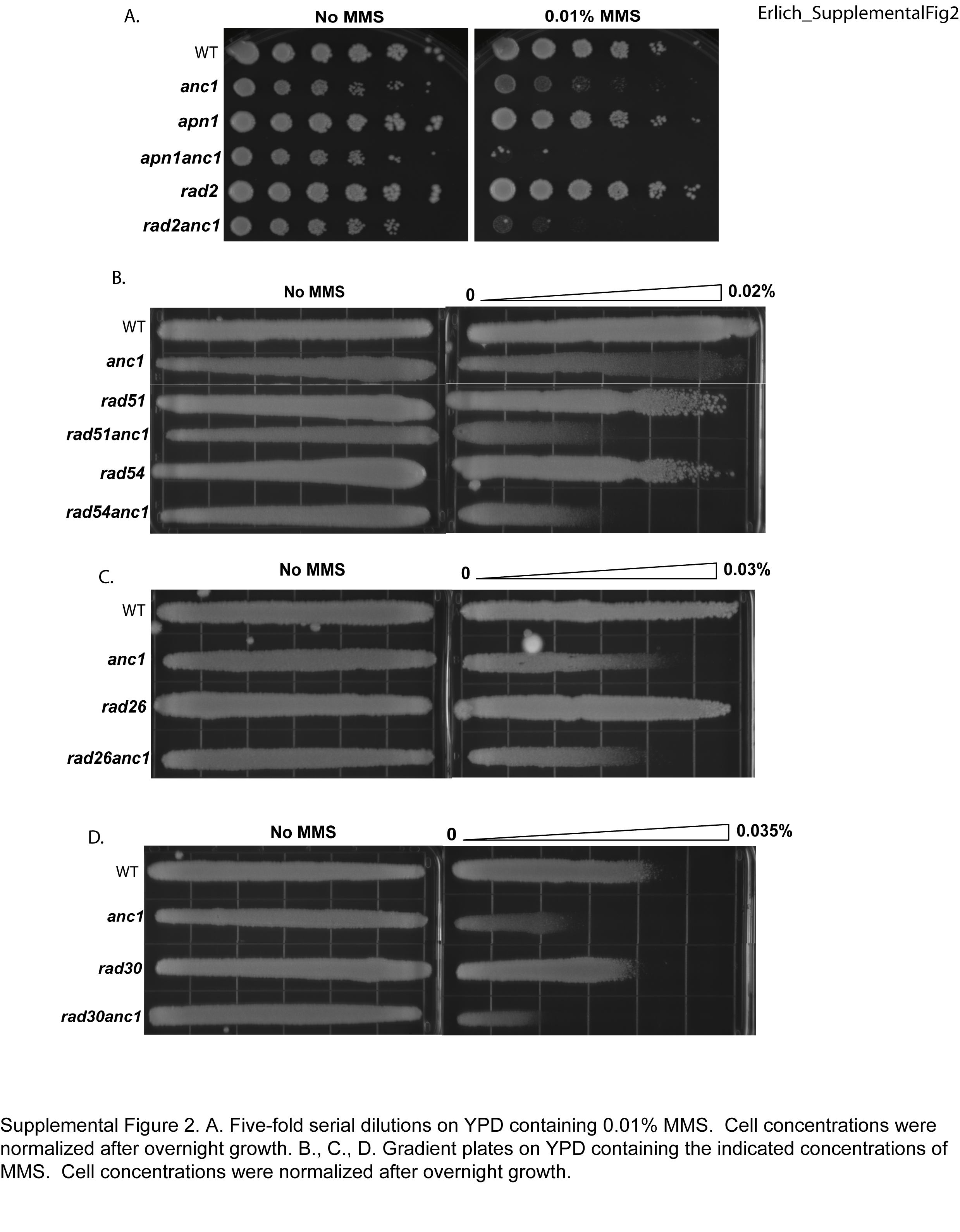

Supplement: Figure S2 — Sensitivity of DNA repair pathway members. A. Five-fold serial dilutions on YPD containing 0.01% MMS. Cell concentrations were normalized after overnight growth. B., C., D. Gradient plates on YPD containing the indicated concentrations of MMS. Cell concentrations were normalized after overnight growth. (23.67 MB DOC) [file pone.0003717.s002.doc]
